# SGLT2 inhibitors and their role in reducing adiposopathy and inflammation in diabetes and non-diabetes CKD patients

**DOI:** 10.1007/s11255-025-04965-6

**Published:** 2025-12-17

**Authors:** Ana Checa-Ros, Owahabanun Joshua Okojie, Nelia Steib, Antonio Bellasi, Pilar Salvador-Martínez, Isabel Fortea, Luis D’Marco

**Affiliations:** 1 Grupo de Investigación en Enfermedades Cardiorenales y Metabólicas, Departamento de Medicina y Cirugía, Facultad de Ciencias de la Salud, Universidad Cardenal Herrera-CEU, CEU Universities, C/Santiago Ramón y Cajal S/N, Alfara del Patriarca, 46115 Valencia, Spain; 2 Aston Institute of Health and Neurodevelopment (AIHN), School of Life and Health Sciences, The Aston Triangle, Aston University, Birmingham, B4 7ET UK; 3 Service of Nephrology, Ospedale Regionale di Lugano, Ospedale Civico, Ente Ospedaliero Cantonale (EOC), Via Tesserete 46, CH-6903 Lugano, Switzerland; 4 Hospital Vithas Consuelo de Valencia. 46007, Valencia, Spain; 5 Grupo de Investigación en Tecnologías de la Encapsulación, Departamento de Medicina y Cirugía, Facultad de Ciencias de la Salud, Universidad Cardenal Herrera-CEU, CEU Universities, 46115 Valencia, Spain; 6 Department of Medicine, Alberta Heart Institute, University of Alberta, Edmonton, AB Canada

**Keywords:** Adiposopathy, SGLT2i, Diabetes, Chronic kidney disease, Inflammation

## Abstract

**Aims:**

This study aims to evaluate differences in adiposopathy and specific inflammatory biomarkers between type 2 diabetes mellitus (T2DM) and non-T2DM patients across various stages of chronic kidney disease (CKD). In addition, it explores potential pathways through which sodium–glucose cotransporter 2 inhibitors (SGLT2i) impact renal outcomes via adipose tissue.

**Materials and methods:**

An observational prospective study was conducted on 143 CKD patients divided into 2 groups: SGLT2i cohort (*n* = 31) and standard-of-care (SoC) cohort (*n* = 112). Clinical and analytical data were collected upon recruitment (T0) as well as after 8 months of follow-up (T8).

**Results:**

At T0, patients under the SGLT2i group showed higher significance for cardiovascular upload versus those in SoC treatment, as well as higher values across several inflammation parameters (IL-6, TNF-α, ferritin). At T8, renal function improved in the SGLT2i group in relation to the SoC, accompanied by a decrease in most inflammatory and adiposopathy biomarkers, mainly leptin. Notably, dapagliflozin use (*n* = 20) was associated with significantly reduced leptin levels and stabilization of TNF-α concentrations vs. SoC at T8.

**Conclusion:**

SGLT2i treatment, and particularly dapagliflozin, modulates both adiposopathy and systemic inflammation while slowing down renal function loss, demonstrating benefits for CKD patients regardless of diabetes status.

**Supplementary Information:**

The online version contains supplementary material available at 10.1007/s11255-025-04965-6.

## Introduction

Chronic kidney disease (CKD) is currently one of the most prevalent non-communicable chronic diseases, affecting more than 10% of the worldwide population. Cardiovascular complications are the leading cause of morbidity and mortality at any stage of CKD [1], mainly secondary to an accelerated atherosclerosis process that likely starts at the early stages of CKD [2]. Both traditional and non-traditional renal risk factors underline the link between CKD and cardiovascular risk. Diabetes mellitus (DM), particularly type 2 (T2DM), arterial hypertension, and obesity have classically been considered as risk factors for CKD, with T2DM being its leading cause in developed countries [3]. Among non-conventional risk factors, particular focus has been put on inflammation, so that CVD could also be linked to the presence of chronic low-grade systemic inflammation in CKD patients as a consequence of the progressive loss of kidney function [4].

Before diabetes initiates, a plethora of metabolic alterations occur, among others: hyperinsulinism, dyslipidemia, fat redistribution and accumulation, as well as adipose tissue dysfunction (*adiposopathy*). Adiposopathy is a process that involves adipose tissue cell remodeling to a pro-inflammatory phenotype, signaling and affecting nearby (paracrine) and distant organs (endocrine). The neuroendocrine and immunometabolism mechanisms underlying adiposopathy in individuals with cardiometabolic and renal diseases are complex and not fully understood [5], although increasing evidence connects adipose tissue dysfunction and CKD progression [6]. Hence, the presence of these two inflammatory conditions may represent a real threat to affected patients, mainly to those at early CKD stages. Therefore, new therapeutic tools targeting adipose tissue may help improve these states [7].

Over the last few years, sodium–glucose cotransporter 2 inhibitors (SGLT2i) have increased substantially in the medical practice due to their documented benefits in cardiorenal and metabolic health. In addition to being used for glycemic control in patients with T2DM, these drugs have other favorable effects, such as weight loss and blood pressure lowering [8]. More recently, they have also been shown to have cardio and renoprotective effects with anti-inflammatory properties [8]. Concerning the latter, the use of these antihyperglycemic agents has been linked to a decrease in pro-inflammatory cytokines and to an improvement in the inflammatory profile in chronic endocrine–metabolic diseases. Hence, these drugs have been positioned as first-line therapy in the management of diabetes and its multiple comorbidities, such as CKD [8, 9].

This study aims at evaluating changes in the adiposopathy and inflammatory biomarkers in patients with and without T2DM diagnosed with mild-to-moderate stages of CKD. Moreover, the study intends to expose evidence on the probable mechanisms, if any, behind the improvement of kidney function after SGLT2i treatments from an adipocentric perspective.

## Methods

### Design

This is a preliminary study from the ADIPO-CKD research project, an ongoing observational prospective cohort study carried out on patients with diverse stages of CKD. The purpose of the ADIPO-CKD project is to establish a link between adiposopathy and renal function markers via the changes observed in adiposopathy and inflammation parameters throughout follow-up treatment with the new antidiabetic medications, such as SGLT2i and glucagon-like peptide-1 receptor agonists (GLP-1RA).

For this study, we compared the changes in adiposopathy and inflammation biomarkers throughout the follow-up of patients receiving SGLT2i against those patients with standard-of-care (SoC) treatment for CKD, with or without T2DM.

### Patient recruitment and follow-up

Patients were recruited from the Nephrology and Internal Medicine Departments at the Virgen del Consuelo Hospital (Valencia, Spain), between January 2023 and October 2024. They were invited to participate in the study if they were: a) ≥ 18 years of age; b) diagnosed with CKD in stages G1, G2, G3a, G3b, and G4, not candidate for dialysis; and c) willing to participate in the study and sign the informed consent. The CKD staging was established according to the estimated glomerular filtration rate (eGFR) as per the current KDIGO guidelines (G1: eGFR ≥ 90 mL/min/1.73 m^2^; G2: 60–89 mL/min/1.73 m^2^; G3a: 45–59 mL/min/1.73 m^2^; G3b: 30–44 mL/min/1.73 m^2^; G4: 15–29 mL/min/1.73 m^2^). Age < 18 years, pregnancy, CKD in stage G5 or G4 candidate for dialysis, neuropsychiatric diseases preventing the patient from understanding the benefits/risks associated with the project, refusal to participate and/or consent revocation were considered as exclusion criteria.

Upon being recruited, patients were divided into two different treatment cohorts based on their compliance with the current clinical practice guidelines for CKD and/or glycemic control in CKD patients [10]:

- The SGLT2i cohort, which included those subjects who met the current criteria to receive treatment with SGLT2i, such as: patients with CKD and T2DM with an eGFR ≥ 20 mL/min/1.73 m^2^; patients with CKD and eGFR ≥ 20 mL/min/1.73 m^2^, accompanied by an urinary albumin-to-creatinine ratio (ACR) ≥ 200 mg/g; patients with CKD and heart failure, irrespective of level of albuminuria; or subjects with CKD and eGFR 20–45 mL/min/1.73 m^2^, with ACR < 200 mg/g.
- The SoC cohort, including those subjects with CKD, with or without T2DM, who met no criteria for treatment with SGLT2i.

Clinical and analytical data collection was performed upon recruitment (T0) and after an average follow-up of 8 months (T8) in both cohorts. For the SGLT2i cohort, T0 represented the measure prior to treatment, whereas T8 was the measure after 8 months of treatment.

### Clinical data collection

Patient electronic medical records from the hospital institutional software (Vithas ONE) were assessed to collect the following demographic and clinical information: age; sex; weight; height; cardiovascular risk factors (arterial hypertension, T2DM, smoking, obesity, hypercholesterolemia); CVD load based on personal history of stroke, myocardial infarct, and peripheral vascular disease, and scored as 0 = none, 1 = personal history of one CVD, 2 = personal history of 2 CVDs, and 3 = personal history of 3 CVDs; other comorbidities; treatment history (angiotensin-converting enzyme inhibitors or ACE inhibitors, angiotensin receptor blockers or ARBs, calcium channel blockers or CCBs, diuretics, nonsteroidal mineralocorticoid receptor antagonists or ns-MRAs, beta-blockers, statins, fibrates, anti-hyperuricemia drugs, vitamin D-based treatments, insulin, metformin, dipeptidyl peptidase 4 inhibitors or DPP4i).

### Analytical data collection

Blood and urine samples were collected from patients in both cohorts at T0 and T8 to measure renal and cardiometabolic function, as well as inflammation and adiposopathy markers, such as tumor necrosis factor-alpha (TNF-α), interleukin-6 (IL-6), C-reactive protein (CRP), ferritin, and leptin. Blood samples were obtained after 8 to 12 h of fasting and a 15-min resting period. Upon blood clot formation, they were centrifuged at 3,500 rpm for 10 min to obtain the serum. First morning urine samples were collected from patients. Laboratory assays involved potentiometry and kinetic colorimetric/photometric analyses on an automated analyzer (ROCHE® model c311; Roche Diagnostics, Barcelona, Spain). After processing, samples were stored at a temperature between – 16 °C and – 28 °C in the Laboratory Unit at the Virgen del Consuelo Hospital.

### Statistics

The Shapiro–Wilk test was used to confirm the parametric distribution of variables. Descriptive data were presented as mean and standard deviation (SD), or as median and interquartile range (IQR), alternatively. Comparisons between the SGLT2i and the SoC groups (inter-group comparison) were performed via: 2 × 2 contingency tables and Chi-square tests of independence (Χ^2^) for categorical clinical and demographic data; and independent samples Student’s t-tests (or their non-parametric alternative Mann–Whitney *U* test) for both analytical values and numerical clinical and demographic characteristics. Paired samples T-test (or their non-parametric version Wilcoxon signed rank test) was run for intragroup comparisons of analytical values before and after treatment (T0 vs T8). Statistical significance was set for a *p* value < 0.05.

The JASP© software version 0.19.3 (The JASP Team, 2018, Amsterdam, The Netherlands) was used to perform the statistical analyses.

### Ethical aspects

The research was conducted following the Declaration of Helsinki as revised in 2013. Authorization was gathered from the Virgen del Consuelo Hospital (code: 23.70), as well as ethical approval from the Biomedical Research Ethics Committee at CEU Cardenal Herrera University (approval code: CEEI23/424). The informed consent was obtained from all patients before being included in the study.

## Results

### Demographic and clinical characteristics of participants

Our sample was composed of 143 patients. Thirty-one of them (21.68%) met criteria for treatment with SGLT2i (SGLT2i cohort), whereas the remaining 112 patients (78.32%) were under SoC treatment.

There was a slight majority of males (58.06%) in the SGLT2i group versus a slight predominance of females (55.36%) in the SoC cohort. SGLT2i cohort patients were older (70 (± 13.75) years in the SGLT2i group vs 65 (± 18.50) years in the SoC group; *p* ~ *0.063*); their body mass index (BMI) was also slightly higher, with no significant differences between the groups (27.86 (± 4.80) kg/m^2^ in the SGLT2i cohort vs 26.60 (± 6.96) kg/m^2^ in the SoC group; *p* ~ *0.597*).

The percentages of patients with cardiovascular risk factors, such as T2DM, hypertension, and smoking, were significantly higher in the SGLT2i group (*p* < *0.011* for hypertension; *p* < *0.001* for T2DM; *p* < *0.036* for smoking). Consequently, the proportion of patients with a CVD load score ≥ 1 was also significantly higher in the SGLT2i cohort (*p* < *0.013*). Although the distribution of patients across CKD severity grades showed no significant differences between the groups (*p* ~ *0.084)*, more than half of the patients in the SGLT2i cohort (58.17%) were classified as G3a and G3b, whereas most patients under SoC (59.82%) were diagnosed with CKD G1 and G2. By the same token, the usage of statins and classical antidiabetic medications significantly predominated in the SGLT2i group: *p* < *0.016* for statins, *p* < *0.001* for metformin, *p* < *0.011* for DPP4i, and *p* < *0.001* for insulin. No significant differences were observed for the usage of other treatments, such as ACE inhibitors, ARBs, CCBs, diuretics, ns-MRAs, fibrates, allopurinol, or vitamin D. The intake of beta-blockers was significantly higher in the SGLT2i cohort (*p* < *0.047*), as well as the percentage of patients on paricalcitol (*p* < *0.045*).

The comparisons of demographic and clinical data between the groups are shown in Table 1.

**Table 1:** Comparisons of demographic and clinical data between patient cohorts (*SGLT2i vs SoC*)

Variables	SGLT2i cohort	SoC cohort	*p*
No of patients (%)	31 (21.68)	112 (78.32)	–
Sex: males (%)	18 (58.06)	50 (44.64)	~ 0.170†
Age (years): median (IQR)	70 (13.75)	65 (18.50)	~ 0.063^‡^
BMI (kg/m^2^): mean (SD)	27.86 (4.80)	26.60 (6.96)	~ 0.597^§^
Blood pressure (mmHg): mean (SD)	95.28 (10.72)	95.94 (10.99)	~ 0.852^§^
Patients with hypertension: *n* (%)	25 (80.64)	62 (55.36)	** < *0.011***†
Patients with T2DM: *n* (%)	11 (35.48)	7 (6.25)	** < *0.001***†
Patients with ADPKD: *n* (%)	2 (6.45)	12 (10.71)	~ 0.480†
Smoking: *n* (%)	6 (19.35)	10 (8.92)	** < *0.036***†
CKD grades: *n* (%) G1 G2 G3a G3b G4	2 (6.45) 9 (29.03) 8 (25.81) 10 (32.36) 1 (3.22)	21 (18.75) 46 (41.07) 13 (11.61) 26 (23.21) 1 (0.89)	~ 0.084† ~ 0.084 ~ 0.084 ~ 0.084 ~ 0.084
CVD load: *n* (%) MI Stroke Peripheral vascular disease	9 (29.03) 4 (12.90) 5 (16.13)	8 (7.14) 7 (6.25) 5 (4.46)	** < *0.001***† ~ 0.219 ** < *0.024***
ACE inhibitors: *n* (%)	6 (19.35)	15 (13.39)	~ 0.407†
ARBs: *n* (%)	16 (51.62)	37 (33.03)	~ 0.058†
CCBs: *n* (%)	8 (25.80)	22 (19.64)	~ 0.456†
Diuretics: *n* (%)	12 (38.71)	33 (29.46)	~ 0.327†
Beta-blockers: *n* (%)	9 (29.03)	16 (14.28)	** < *0.047***†
ns-MRAs: *n* (%)	3 (9.67)	6 (5.36)	~ 0.381†
Metformin: *n* (%)	9 (29.03)	7 (6.25)	** < *0.001***†
DPP4i: *n* (%)	5 (16.13)	4 (3.57)	** < *0.011***†
Insulin: *n* (%)	4 (12.90)	1 (0.89)	** < *0.001***†
Statins: *n* (%)	20 (64.52)	45 (40.18)	** < *0.016***†
Fibrates: *n* (%)	2 (6.45)	3 (2.68)	~ 0.333†
Allopurinol: *n* (%)	7 (22.58)	13 (11.61)	~ 0.119†
Vitamin D and Calcitriol: *n* (%)	2 (6.45)	9 (8.03)	~ 0.056†
Paricalcitol: *n* (%)	4 (12.90)	4 (3.57)	** < *0.041***†

Statistical comparisons performed through: ^†^Chi-square test of independence

^‡^Mann–Whitney *U* test

^§^Independent samples Student’s *t* test

*SGLT2i* sodium–glucose cotransporter 2 inhibitors, *SoC* standard of care, *%* percentage, *IQR* interquartile range, *BMI* body mass index, *SD* standard deviation, *n* number of patients, *T2DM* type 2 diabetes mellitus, *ADPKD* autosomal-dominant polycystic kidney disease, *CKD* chronic kidney disease, *G1* grade 1 of chronic kidney disease, *G2* grade 2 of chronic kidney disease, *G3a* grade 3a of chronic kidney disease, *G3b* grade 3b of chronic kidney disease, *G4* grade 4 of chronic kidney disease, *CVD* cardiovascular disease, *MI* myocardial infarction, *ACE* angiotensin-converting enzyme, *ARBs* angiotensin receptor blockers, *CCBs* calcium channel blockers, *ns-MRAs* nonsteroidal mineralocorticoid receptor antagonists, *DPP4i* dipeptidyl peptidase 4 inhibitors

### Analytical characteristics of participants

#### Baseline biochemical values

Analytical values at T0, before treatment adjustment and the initiation of SGLT2i, are shown in Table 2. Reasonably expected findings were observed when comparing parameters of renal and cardiometabolic function between the groups: significantly higher levels of glucose (*p* < *0.006*), urea (*p* < *0.001*), creatinine (*p* < *0.001*), microalbuminuria (*p* < *0.001*), and urinary ACR (*p* < *0.001*) were found in the patients from the SGLT2i group, which translated into a significantly lower mean eGFR in comparison with the SoC cohort (*p* < *0.002*). Low-density lipoprotein cholesterol (LDL-cholesterol) mean values were, however, significantly increased in the SoC group (*p* < *0.046*). Baseline values of adiposopathy and inflammation markers showed heterogeneous but not statistically significant differences between the groups: leptin levels were lower in the SGLT2i group, in parallel with CRP concentrations, and in contrast to mean values of ferritin, TNF-α, and IL-6, which were elevated in comparison with the SoC cohort.

**Table 2:** Comparisons of basal analytical data (T0) and after treatment (T8) between patient cohorts (*SGLT2i vs SoC*)

Variables	T0			T8			
	SGLT2i cohort	SoC cohort	*p*	SGLT2i cohort		SoC cohort	*p*
Hematocrit (%): mean (SD)	44.53 (4.65)	43.30 (4.56)	~ 0.196^†^	44.34 (3.96)		44.15 (4.44)	~ 0.873^†^
Glucose (mg/dL): median (IQR)	106.50 (25.50)	97 (16)	** < *0.006***^‡^	106 (29)		99 (15)	** < *0.029***^‡^
Urea (mg/dL): median (IQR)	50 (18)	38 (20)	** < *0.001***^‡^	46 (17.50)		41.50 (18.25)	~ 0.072^‡^
Creatinine (mg/dL): mean (SD)	1.27 (0.36)	1.01 (0.31)	** < *0.001***^†^	1.25 (0.32)		1.02 (0.30)	** < *0.003***^†^
eGFR (mL/min/1.73 m^2^): mean (SD)	56.11 (17.67)	70.75 (23.03)	** < *0.002***^†^	58.02 (16.64)		67.77 (19.61)	~ 0.153^†^
Uric acid (mg/dL): mean (SD)	5.41 (1.47)	5.64 (1.40)	~ 0.439^†^	5.55 (1.27)		5.66 (1.52)	~ 0.780^†^
Albumin (g/L): mean (SD)	4.59 (0.40)	5.22 (6.12)	~ 0.634^†^	4.62 (0.34)		4.46 (0.30)	~ 0.634^†^
Calcium (mg/dL): mean (SD)	9.71 (0.46)	9.73 (0.44)	~ 0.834^†^	9.70 (0.65)		9.70 (0.30)	~ 0.986^†^
Phosphate (mg/dL): mean (SD)	3.81 (0.58)	3.58 (0.55)	~ 0.090^†^	3.79 (0.55)		3.69 (0.51)	~ 0.510^†^
LDL-cholesterol (mg/dL): mean (SD)	86.68 (38.08)	105.42 (43.60)	** < *0.046***^†^	86.38 (27.71)		108 (34.03)	** < *0.019***^†^
Triglycerides (mg/dL): median (IQR)	103 (56)	97 (56)	~ 0.651^‡^	104 (44)		93 (46)	~ 0.524^‡^
Glycated hemoglobin (%): mean (SD)	6 (0.76)	5.72 (0.44)	** < *0.026***^†^	6.24 (0.94)		5.76 (0.56)	** < *0.018***^†^
iPTH (pg/mL): mean (SD)	54.97 (20.45)	47.34 (23.12)	~ 0.753^†^	66.02 (34.05)		60.58 (39.92)	~ 0.675^†^
Ferritin (ng/mL): mean (SD)	168.19 (165.14)	139.77 (99.26)	~ 0.357^†^	133.14 (119.31)		147.70 (96.36)	~ 0.706^†^
Leptin (ng/mL): mean (SD)	19.80 (16.90)	24.30 (29.22)	~ 0.467^†^	15.78 (12.46)		29.71 (25.64)	** < *0.040***^†^
TNF-α (pg/mL): mean (SD)	11.55 (8.83)	8.44 (3.12)	~ 0.054^†^	9.68 (4.14)		7.97 (1.68)	~ 0.051^†^
IL-6 (pg/mL): mean (SD)	5.45 (6.91)	3.89 (2.07)	~ 0.097^†^	4.80 (3.26)		3.25 (1.51)	~ 0.058^†^
CRP (mg/L): mean (SD)	2.49 (3.65)	4.77 (11.48)	~ 0.323^†^	2.11 (1.82)		3.29 (5.80)	~ 0.417^†^
MA (mg/L): mean (SD)	97.41 (182.14)	16.33 (39.97)	** < *0.001***^†^	55.25 (94.21)		21.45 (56.54)	~ 0.085^†^
Urinary ACR (mg/g): mean (SD)	169.61 (302.30)	23.09 (82.97)	** < *0.001***^†^	94.84 (193.93)	25.17 (60.34)		** < *0.035***^†^

Statistical comparisons performed through: ^†^Independent Samples Student’s *t* test

^‡^Mann–Whitney *U* test

*SGLT2i* sodium–glucose cotransporter 2 inhibitors, *SoC* standard of care, *IQR* interquartile range, *SD* standard deviation, *eGFR* estimated glomerular filtration rate, *LDL-cholesterol* low-density lipoprotein cholesterol, *%* percentage, *iPTH* intact parathyroid hormone, *TNF-α* tumor necrosis factor-alpha, *IL-6* interleukin-6, *CRP* C-reactive protein, *MA* microalbuminuria, *ACR* albumin-to-creatinine ratio

#### Biochemical values after follow-up

A follow-up on the adiposopathy and inflammatory profile of patients, together with their main renal and cardiometabolic variables, was performed after 8.10 (± 2.11) months of treatment (T8), as also shown in Table 2. Most patients in the SGLT2i cohort received dapagliflozin (*n* = 20, 64.52%), whereas the rest of them received different gliflozins (35.48%), such as empagliflozin or canagliflozin. A modest decrease in BMI from 27.86 (± 4.80) kg/m^2^ to 26.28 (± 4.10) kg/m^2^ was observed in the SGLT2i group, versus an increase from 26.60 (± 6.96) kg/m^2^ to 27.58 (± 7.10) kg/m^2^ in the SoC group, with no significant differences between the groups. No considerable differences were observed in cardiometabolic function parameters after treatment follow-up. Variables of renal function improved in the SGLT2i group at T8, characterized by a non-significant but remarkable decrease in microalbuminuria (from 97.41 mg/L to 55.25 mg/L [*p* ~ 0.798]) and urinary ACR (from 169.91 mg/g before treatment to 94.84 mg/g after treatment [*p* ~ 0.406]), in contrast to an overall worsening of renal function in the SoC cohort. As a result, the significant differences observed in the eGFR between the groups at T0 disappeared after treatment (from 56.11 mL/min/1.73 m^2^ in the SGLT2i cohort vs. 70.75 mL/min/1.73 m^2^ [*p* < *0.002*] in the SoC group, to 58.02 mL/min/1.73 m^2^ vs. 67.77 mL/min/1.73 m^2^, respectively [*p* ~ 0.153]). These changes were accompanied by modifications in adiposopathy and inflammatory markers within the two groups. In the SGLT2i group, a non-significant decrease in leptin levels was observed (from 19.80 ng/mL to 15.78 ng/mL [*p* ~ 1.000]), contrary to the augmented leptin concentrations observed in the SoC after follow-up (from 24.30 ng/mL to 29.71 ng/mL [*p* ~ 1.000]). When comparing between the groups, leptin concentrations at T8 were significantly lower in patients under SGLT2i treatment than in patients receiving SoC (*p* < *0.040*). Regarding the remaining systemic inflammatory markers, the most outstanding result was a decrease in ferritin concentrations in the SGLT2i group (from 168.19 ng/mL to 133.14 ng/mL [*p* ~ 0.154]), in contrast to an increase in this parameter in the SoC cohort (from 139.77 ng/mL to 147.70 ng/mL [*p* ~ 1.000]). IL-6 levels slightly decreased in the SGLT2i group (from 5.45 pg/mL to 4.80 pg/mL [*p* ~ 0.379]), whereas they remained stable among patients under SoC, with no significant differences between the groups. A non-significant reduction in TNF-α concentrations was observed in both groups, more remarkably in the SGLT2i one. CRP concentrations, however, remained almost similar in both groups.

#### Comparisons between SoC and the most common SGLT2i (dapagliflozin)

Given that no statistically significant differences between the groups were observed for most inflammation and adiposopathy variables after treatment, we decided to compare these values and the eGFR at T8 between SoC patients (*n* = 112) and those receiving dapagliflozin (*n* = 20), as the most administered SGLT2i in our cohort. The remaining 11 patients on SGLT2i received empagliflozin (*n* = 9) and canagliflozin (*n* = 2).

The mean eGFR moderately decreased from 70.75 (± 23.03) mL/min/1.73 m^2^ to 67.77 (± 19.61) mL/min/1.73 m^2^ after SoC treatment, as seen in Tables 1 and 2. However, this decrease was not observed after treatment with dapagliflozin (from 51.07 (± 12.56) mL/min/1.73 m^2^ to 54.78 (± 15.84) mL/min/1.73 m^2^) (**Supplementary Fig. 1**).

**Fig. 1:**
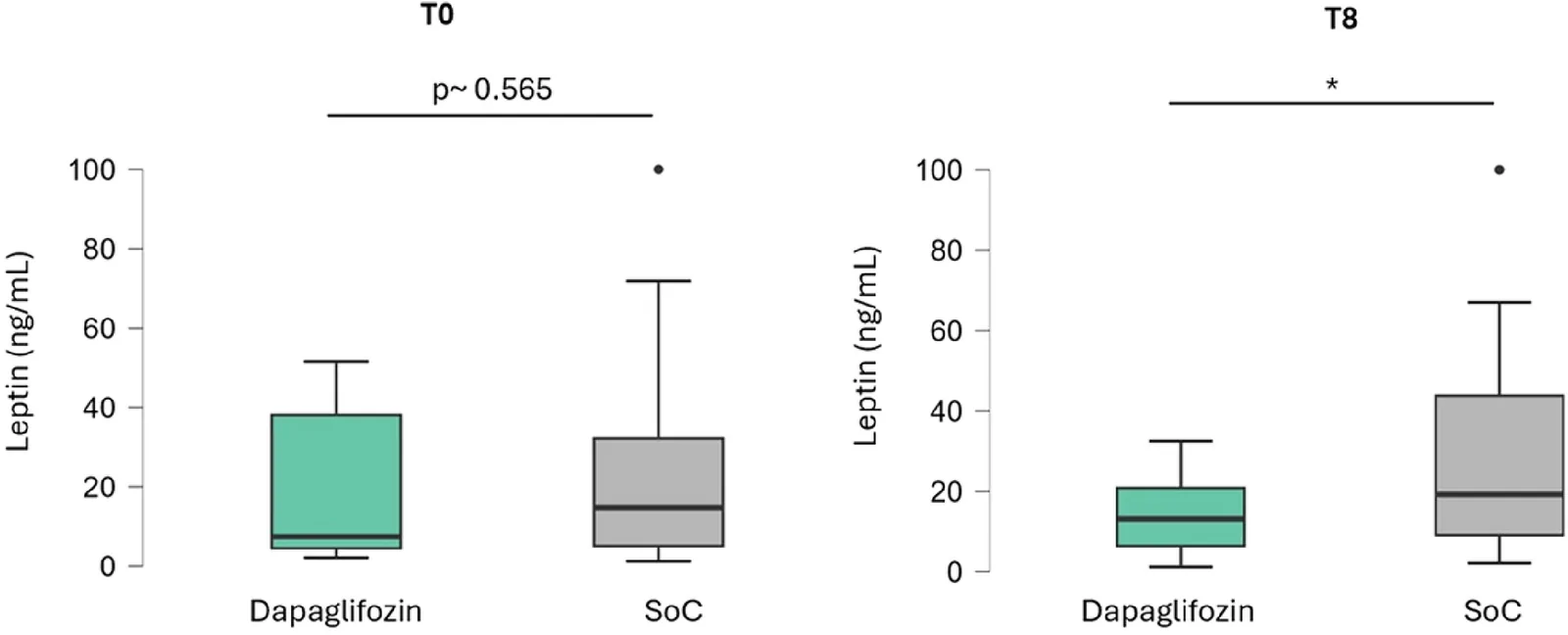
Comparison of leptin levels between patients on dapagliflozin and those receiving standard of care (SoC). Outliers are labeled *p < *0.05*

As aforementioned, the mean value of leptin as a marker of adiposopathy increased from 24.30 (± 29.22) ng/mL to 29.71 (± 25.64) ng/mL in the SoC group. Nevertheless, dapagliflozin use was associated with a decrease in leptin levels from 19.43 (± 19.35) ng/mL to 14.28 (± 10.25) ng/mL, so that leptin values in the dapagliflozin group were significantly lower than in the SoC cohort at T8 (*p* < *0.021*) (Fig. 1).

**Fig. 2:**
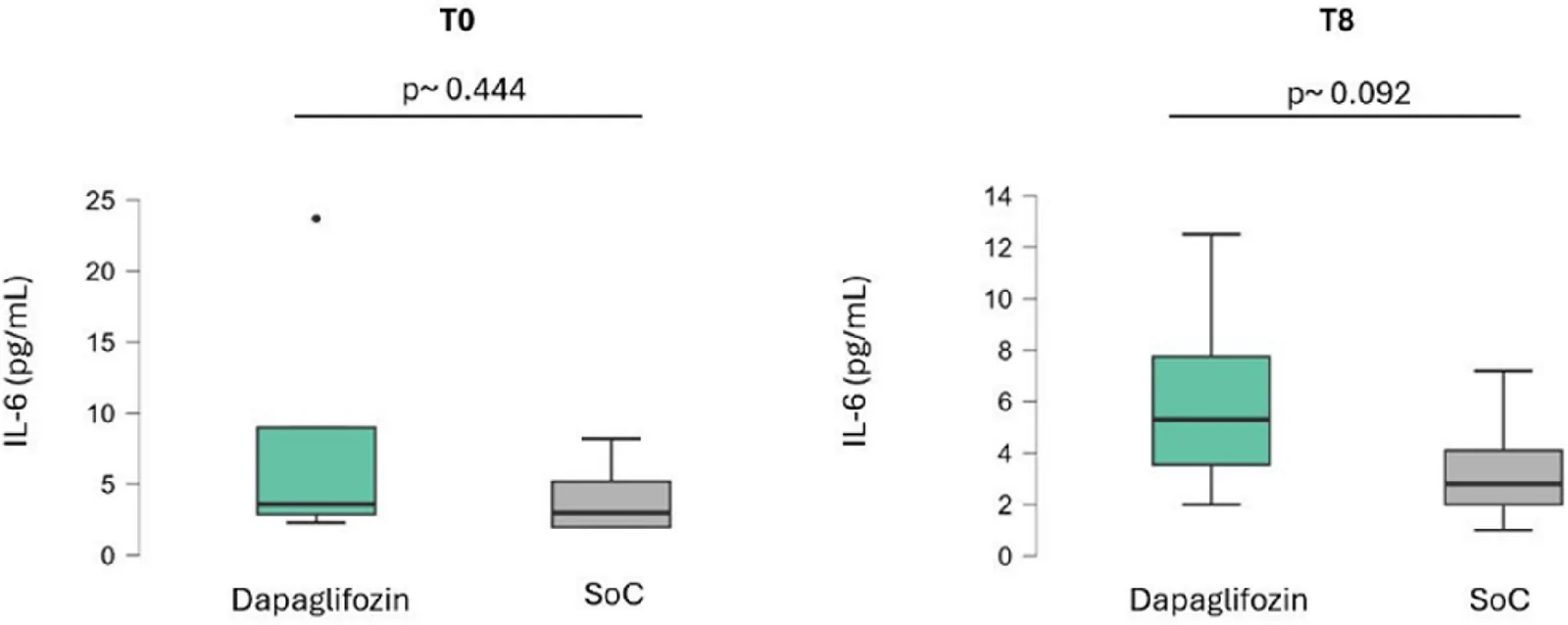
Comparison of interleukin-6 (IL-6) levels between patients on dapagliflozin and those receiving standard of care (SoC). Outliers are labeled

**Fig. 3:**
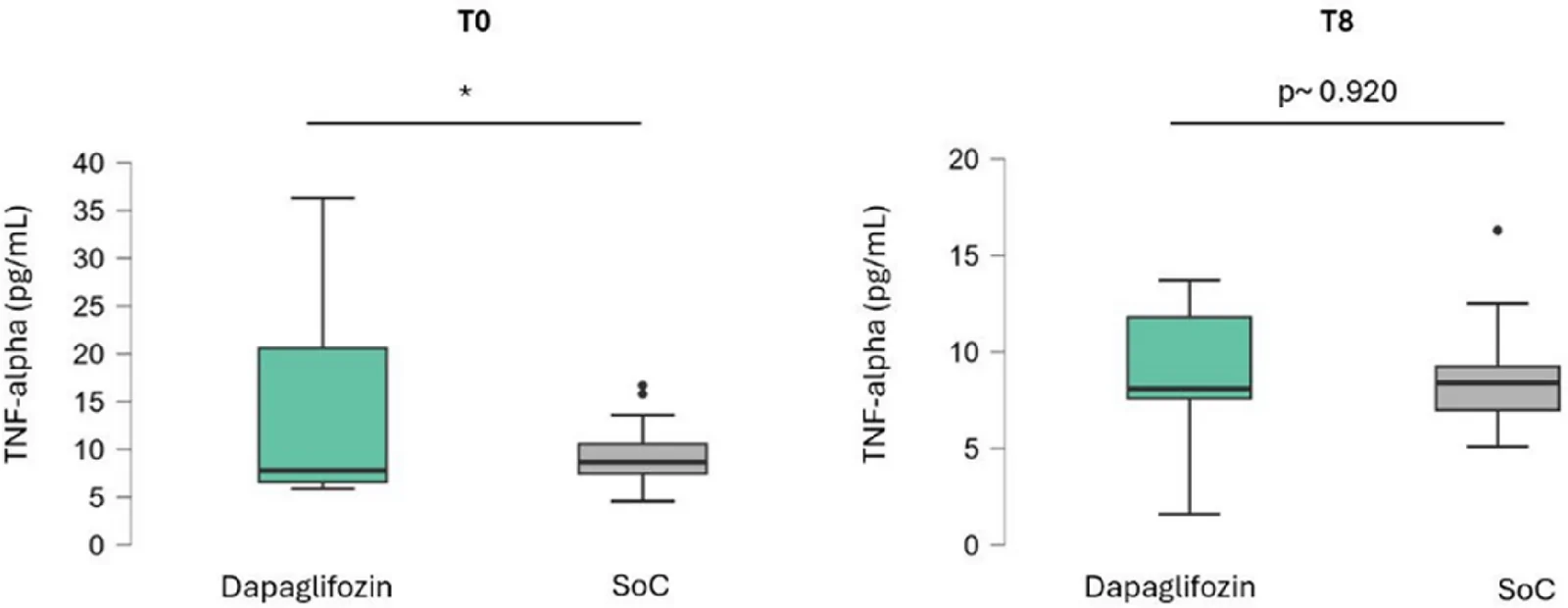
Comparison of TNF-alpha levels between patients on dapagliflozin and those receiving standard of care (SoC). Outliers are labeled. * p < *0.05*

Regarding inflammatory markers, IL-6 also followed a decreasing pattern after treatment with dapagliflozin, diminishing from a mean of 8.30 (± 10.29) pg/mL to 6.06 (± 3.73) pg/mL, although IL-6 concentrations in the SoC cohort were still lower at T8 (*p* ~ 0.092) (Fig. 2). A reduction trend was also observed for TNF-α, so that values at T0 were significantly higher in the dapagliflozin group (14.91 [± 12.47] pg/mL vs. 8.44 [± 3.12] pg/mL in the SoC [*p* < *0.012*]), whereas these significant differences disappeared after treatment (8.77 [± 4.08] pg/mL with dapagliflozin vs. 7.97 [± 1.68] pg/mL with SoC) (p ~ 0.920) (Fig. 3). Only minor differences in CRP levels were found in both groups at T0 and T8 (from 3.57 (± 4.62) mg/L to 2.43 (± 2.03) mg/L after dapagliflozin treatment vs a change from 4.77 (± 11.48) mg/L to 3.29 (± 5.80) mg/L in the SoC group) (**Supplementary Fig. 2**).

A similar decreasing trend in adiposopathy and inflammatory markers (leptin and TNF-α, in particular), accompanied by an improvement in eGFR, was also observed among those patients under the other SGLT2is (mainly empagliflozin). No differences were observed between these two SGLT2is (empagliflozin and canagliflozin) probably due to just only two patients receiving canagliflozin.

## Discussion

Our study demonstrates two pivotal findings: (1) according to the results in leptin levels, Dapagliflozin improves adiposopathy parameters in mild-to-moderate CKD patients, irrespective of T2DM status; and (2) it reduces inflammatory biomarkers, notably TNF-α. In addition, as anticipated, SGLT2i attenuated CKD progression, evidenced by keeping eGFR stable and decreasing albuminuria after 8 months of treatment.

The weight loss effects of SGLT2i are well documented in T2DM, with clinical trials and meta-analyses reporting an average reduction of 1.5–3 kg in body weight, primarily within the first 24 weeks of treatment [10–19]. Notably, the EMPA-REG OUTCOME trial demonstrated that empagliflozin reduced visceral adiposity in patients with diabetes, independent of glycemic control [20]. However, our study observed only a non-significant BMI decrease, in contrast with these reports. This discrepancy may concern the population differences. Our cohort included both T2DM and non-diabetes CKD patients, whereas most trials focused on T2DM. Renal dysfunction itself alters adipose tissue metabolism, potentially blunting SGLT2i effects. Moreover, compensatory hyperphagia, as seen in the DAPA-HF trial, by which caloric intake increases, may offset urinary glucose excretion, attenuating weight loss [15]. Mechanistically, SGLT2i shifts the insulin/glucagon balance, promoting lipolysis via glucagon-mediated pathways [16, 17, 21]. However, genetic polymorphisms (e.g., SLC5A2 variants) and concomitant medications (e.g., insulin) can modulate these, underscoring the need for personalized approaches [18, 19]^.^

There is evidence that Dapagliflozin exerts pleiotropic metabolic benefits, including the modulation of leptin secretion [22]. Leptin, an adipokine critical for energy homeostasis, is frequently elevated in obesity and T2DM, contributing to leptin resistance and metabolic dysfunction [22]. In line with our results, clinical studies demonstrate that Dapagliflozin significantly lowers circulating leptin levels, primarily through its catabolic effects on adipose tissue. Xu et al. (2021) reported a 23% reduction in serum leptin in T2DM patients after 6 months of therapy, paralleling decreases in visceral adiposity (measured by MRI) and improved insulin sensitivity (HOMA-IR; *p* < 0.01). This suggests that Dapagliflozin's promotion of lipolysis and fat mass reduction directly attenuates hyperleptinemia, thereby restoring leptin sensitivity [23].

Beyond adiposity reduction, Dapagliflozin may suppress leptin via anti-inflammatory pathways [23]. Elevated leptin in metabolic syndrome is driven by pro-inflammatory cytokines (e.g., TNF-α, IL-6), which stimulate leptin gene expression in adipocytes. Jojima et al. [24] observed that Dapagliflozin treatment in T2DM patients reduced TNF-α by 34% and leptin by 28% (*p* < 0.05), independent of weight loss. This aligns with in vitro data showing SGLT2i downregulates nuclear factor-kappa beta (NF-κB) signaling in adipocytes [25]. The dual modulation of adipose tissue mass and inflammation emphasizes Dapagliflozin’s role in ameliorating leptin-associated metabolic dysregulation, potentially explaining its cardiorenal benefits in large-scale trials (e.g., DAPA-HF and DECLARE-TIMI) [16].

Our analysis reveals that TNF-α is the inflammatory marker that undergoes a statistically significant reduction following treatment. This finding aligns with a growing body of evidence suggesting that SGLT2 inhibitors exert potent effects on TNF-α pathways [26, 27]. For instance, preclinical models demonstrate that empagliflozin directly reduces TNF-α expression in macrophages and adipose tissue, a key source of this cytokine [26–28]. Although our results did not show significant changes in IL-6, CRP, or ferritin, the specific reduction in TNF-α highlights a targeted anti-inflammatory mechanism. This divergence from the EMPA-TROPISM trial (which reported CRP reductions) may stem from tissue-specific effects; SGLT2Is may preferentially target pathways upstream of TNF-α production [29]. Furthermore, the unique immune dysregulation in CKD, where impaired cytokine clearance can mask systemic effects, might explain the lack of significance in other markers [30–32]. Notably, while the DAPA-CKD trial reported lower IL-6 in diabetic CKD patients on dapagliflozin [33], our study in a non-diabetic CKD cohort suggests that the anti-TNF-α effect is a fundamental and perhaps more consistent property of this drug class, extending its benefits beyond a diabetic population—a novel insight with significant clinical relevance.

SGLT2i induces white adipose tissue remodeling, shifting it from a pro-inflammatory and metabolically stressed state to a more oxidative and energy-efficient state [34]. Preclinical studies suggest that SGLT2i promotes white adipose tissue browning via M2 macrophage-derived cytokines (e.g., IL-10) [26, 35], with the consequent enhanced mitochondrial content and upregulation in the expression of thermogenic markers, such as the uncoupled protein-1 (UCP-1) and peroxisome proliferator-activated receptor gamma coactivator 1-alpha (PGC-1α) [36, 37]. In humans, the CANTATA-SU trial found that canagliflozin upregulated mitochondrial genes (e.g., PGC-1α) in the adipose tissue, mirroring our hypothesis [38]. However, clinical evidence remains scarce. Unexpectedly, a study on murine models with dapagliflozin reported no upregulation of uncoupled protein-1 (UCP-1) despite increased sympathetic activity, highlighting drug- and species-specific differences [39]. Conversely, empagliflozin increased brown adipose tissue activation in obese mice, suggesting class effects may vary by agent [40]. This browning process is accompanied by increased energy expenditure, improved insulin sensitivity, and reduced adipose tissue fibrosis via suppression of the leptin-induced JAK2-STAT3-ROS pathway, counteracting adiposopathy even without substantial weight loss [37, 41].

Regarding CKD-specific implications and novelty, our study is among the first to link SGLT2i (Dapagliflozin) with adiposopathy improvement in non-diabetes CKD, a population often excluded from the main metabolic trials. This aligns with post-hoc analyses of CREDENCE (canagliflozin in CKD), showing renal benefits regardless of diabetes status [42]. However, the lack of significant BMI changes in our cohort contrasts with trials on CKD with diabetes (e.g., EMPA-KIDNEY), suggesting renal function may modulate metabolic responses [43].

Although the present study focuses on SGLT2i, the ongoing ADIPO-CKD project also includes a cohort of patients treated with GLP-1RA. This medication is reported to induce more pronounced weight loss (predominantly fat mass) than SGLT2i, also accompanied by a reduction of pro-inflammatory adipokines and adipose tissue browning [44–46]. Head-to-head combination studies comparing SGLT2i and GLP-1RA in CKD patients with and without diabetes will be crucial to determine the relative and potentially synergistic contributions of each drug class.

Our study findings have several limitations to consider. Key limitations include the small sample size and types in the SGLT2i group, short follow-up period, and absence of adipose tissue biopsies to confirm mechanistic insights. Thus, future studies should assess long-term effects on adipose tissue morphology, compare SGLT2i subtypes and doses, and finally, explore combinatorial therapies targeting hyperphagia or mitochondrial dysfunction.

In conclusion, our finding supports that treatment with SGLT2i (specifically dapagliflozin) positively influences adiposopathy and inflammatory parameters, such as leptin, and TNF-α, with the known effect on reducing microalbuminuria with kidney function preservation. Moreover, our novel findings concern the ability of this drug to improve these biomarkers in non-diabetes CKD populations. Consequently, dapagliflozin’s effects could improve adipose tissue dysfunction beyond the presence of obesity and/or T2DM. Further studies are needed to validate these findings.

## Supplementary Information

Below is the link to the electronic supplementary material.

Supplementary file1 Comparison of mean eGFR values between patients on dapagliflozin and those receiving standard of care (SoC). Outliers are labeled **p<0.01 (TIFF 109 KB)

Supplementary file2 Comparison of C-reactive protein (CRP) levels between patients on dapagliflozin and those receiving standard of care (SoC). Outliers are labeled. (TIFF 88 KB)

## Data Availability

The datasets used and analyzed during the current study are available from the corresponding author upon reasonable request.
