# Correction: SGLT2 inhibitors and their role in reducing adiposopathy and inflammation in diabetes and non-diabetes CKD patients

**DOI:** 10.1007/s11255-026-05028-0

**Published:** 2026-02-16

**Authors:** Ana Checa-Ros, Owahabanun-Joshua Okojie, Nelia Steib, Antonio Bellasi, Pilar Salvador-Martínez, Isabel Fortea, Luis D’Marco

**Affiliations:** 1 Grupo de Investigación en Enfermedades Cardiorenales y Metabólicas, Departamento de Medicina y Cirugía, Facultad de Ciencias de la Salud, Universidad Cardenal Herrera-CEU, CEU Universities, C/Santiago Ramón y Cajal S/N, Alfara del Patriarca, 46115 Valencia, Spain; 2 Aston Institute of Health and Neurodevelopment (AIHN), School of Life and Health Sciences, The Aston Triangle, Aston University, Birmingham, B4 7ET UK; 3 Service of Nephrology, Ospedale Regionale di Lugano, Ospedale Civico, Ente Ospedaliero Cantonale (EOC), Via Tesserete 46, CH-6903 Lugano, Switzerland; 4 Hospital Vithas Consuelo de Valencia. 46007, Valencia, Spain; 5 Grupo de Investigación en Tecnologías de la Encapsulación, Departamento de Medicina y Cirugía, Facultad de Ciencias de la Salud, Universidad Cardenal Herrera-CEU, CEU Universities, 46115 Valencia, Spain; 6 Department of Medicine, Alberta Heart Institute, University of Alberta, Edmonton, AB Canada

**Correction: International Urology and Nephrology** 10.1007/s11255-025-04965-6

In the original version of this article, the given and family names of few author names were incorrectly structured. The correct given and family names are given below.

Given name: Ana; Family name: Checa-Ros

Given name: Owahabanun-Joshua; Family name: Okojie

Given name: Nelia; Family name: Steib

Given name: Antonio; Family name: Bellasi

Given name: Pilar; Family name: Salvador-Martínez

Given name: Isabel; Family name: Fortea

Given name: Luis; Family name: D’ Marco

The original article has been corrected.

