# Supplementary figures and images for: SGLT2 inhibitors and their role in reducing adiposopathy and inflammation in diabetes and non-diabetes CKD patients
Source: Int Urol Nephrol. 2025 Dec 17;58(7):2721–30. doi: 10.1007/s11255-025-04965-6 (PMC13309401; doi:10.1007/s11255-025-04965-6)

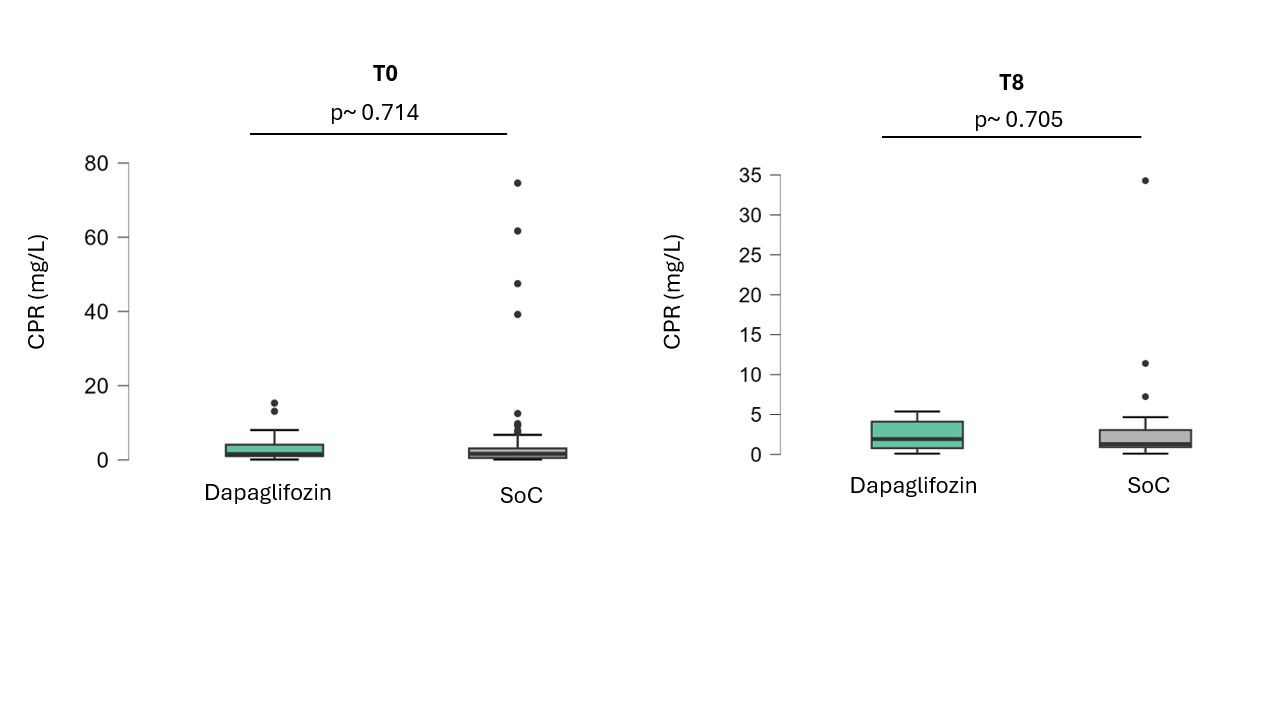

Supplement: Supplementary file 1 — Supplementary file1 Comparison of mean eGFR values between patients on dapagliflozin and those receiving standard of care (SoC). Outliers are labeled **p<0.01 (TIFF 109 KB) [file 11255_2025_4965_MOESM1_ESM.tiff]

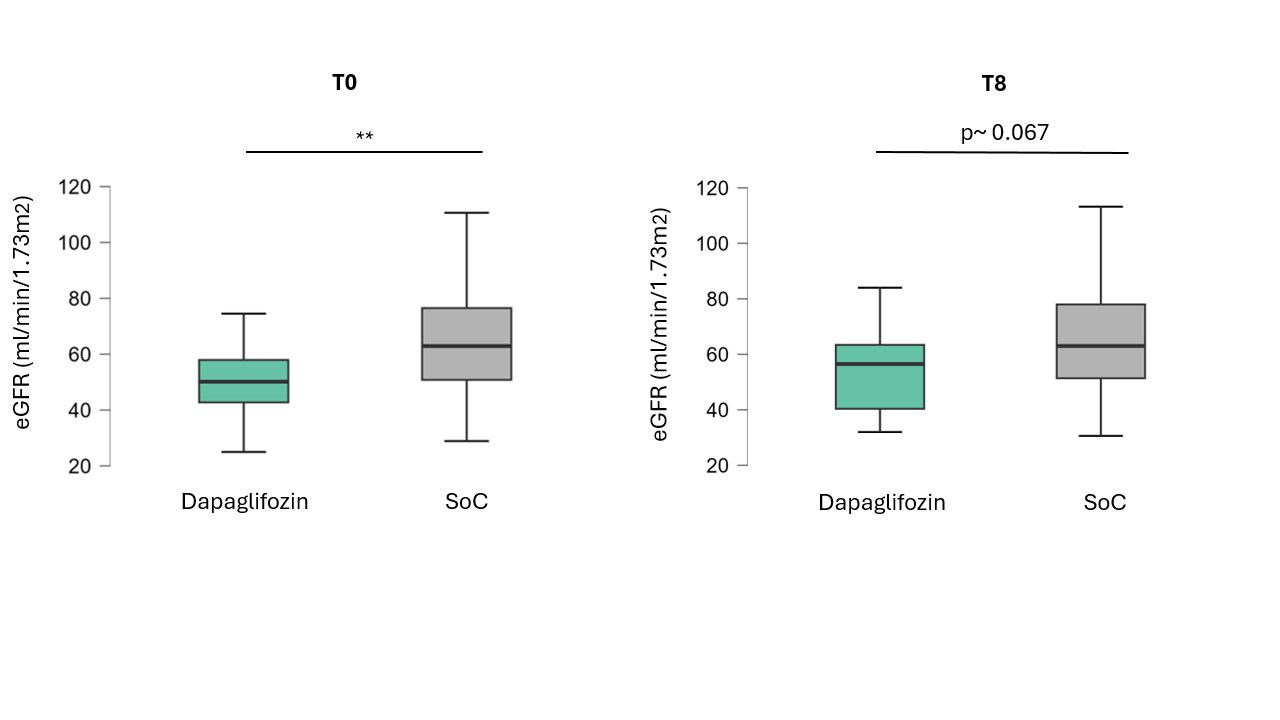

Supplement: Supplementary file 2 — Supplementary file2 Comparison of C-reactive protein (CRP) levels between patients on dapagliflozin and those receiving standard of care (SoC). Outliers are labeled. (TIFF 88 KB) [file 11255_2025_4965_MOESM2_ESM.tiff]
